# Mouse study of combined DNA/protein COVID-19 vaccine to boost high levels of antibody and cell mediated immune responses

**DOI:** 10.1080/22221751.2022.2152388

**Published:** 2022-12-12

**Authors:** Qian He, Shuying Liu, Zhenglun Liang, Shan Lu, Wei Cun, Qunying Mao

**Affiliations:** a National Institute for Food and Drug Control, Beijing, People’s Republic of China; b SYL-Consulting, Thousand Oaks, CA, USA; c Laboratory of Nucleic Acid Vaccines, Department of Medicine, University of Massachusetts Medical School, Worcester, MA, USA; d Institute of Medical Biology, Chinese Academy of Medical Sciences & Peking Union Medical College, Kunming, People’s Republic of China

**Keywords:** SARS-CoV-2, DNA vaccine, protein vaccine, heterologous prime – boost, antibody, T cells

Rapid development of effective vaccines against SARS-CoV-2 has been a major achievement in the global effort of controlling the COVID-19 pandemic [1–5]. However, the levels of protective antibodies in vaccinated human populations dropped quite rapidly with reduced levels of protection against infection or the diseases development [6–8]. Additional booster immunizations after the original vaccine series (such as the twice immunizations with inactivated vaccines) have become necessary to enhance waning immunity against SARS-COV-2 and emerging variants [9,10].

Multiple human studies reported the choice of booster immunization may determine the levels of protective antibody responses [11–13]. For more immunogenic vaccines such as mRNA vaccines, boost immunization with mRNA vaccines are usually able to bring back the high-level of protective antibody responses observed shortly after the first two immunizations [11,12,14]. On the other hand, humans received priming immunization with adenoviral vector-based vaccines showed lower antibody responses after boosting with the same viral vector vaccines than the boost with mRNA vaccines [11,12].

The use of different vaccine modalities for prime and boost was described in literature as the heterologous prime-boost immunization in which the prime and boost steps use different types of vaccines to deliver the same protective antigen [15]. It is well established that the heterologous prime-boost strategies may offer immunologic advantages to elicit potent immune responses and longevity of protection [16,17]. Recent reports further confirmed that the heterologous prime-boost approach is also highly effective for COVID-19 vaccines, such as Ad vector prime – mRNA boost [11,12,18], inactivated prime – protein boost [19] and inactivated prime – aerosolized Ad vector boost [20,21] which were more efficient than prime-boost using the same Ad vector or inactivated COVID-19 vaccines.

In our previous studies, we showed the high immunogenicity of heterologous DNA prime, protein boost HIV vaccines in humans [17,22]. More recently a combination DNA/Protein COVID-19 vaccine developed by us showed high immunogenicity and sterilizing protection in a non-human primate model against the SARS-CoV-2 challenge [23]. In the current study, we aim to test whether this combination DNA/Protein vaccine can also be used as a booster to other types of COVID-19 vaccines.

The mouse model was used in this pilot study. Heterologous boost with the combination DNA/Protein vaccine (DP) was applied to animals who were first primed with either of the three COVID-19 vaccines: inactivated SARS-CoV-2 virus vaccine (IV), chimpanzee adenovirus vector vaccine encoding full length S antigen (ChAd-S) or mRNA vaccine encoding receptor binding domain (RNA-RBD) (Figure 1(a)). All vaccines used in this study were targeting the ancestral SARS-CoV-2 strain and still trial vaccines in the laboratory stage.

**Figure 1.**
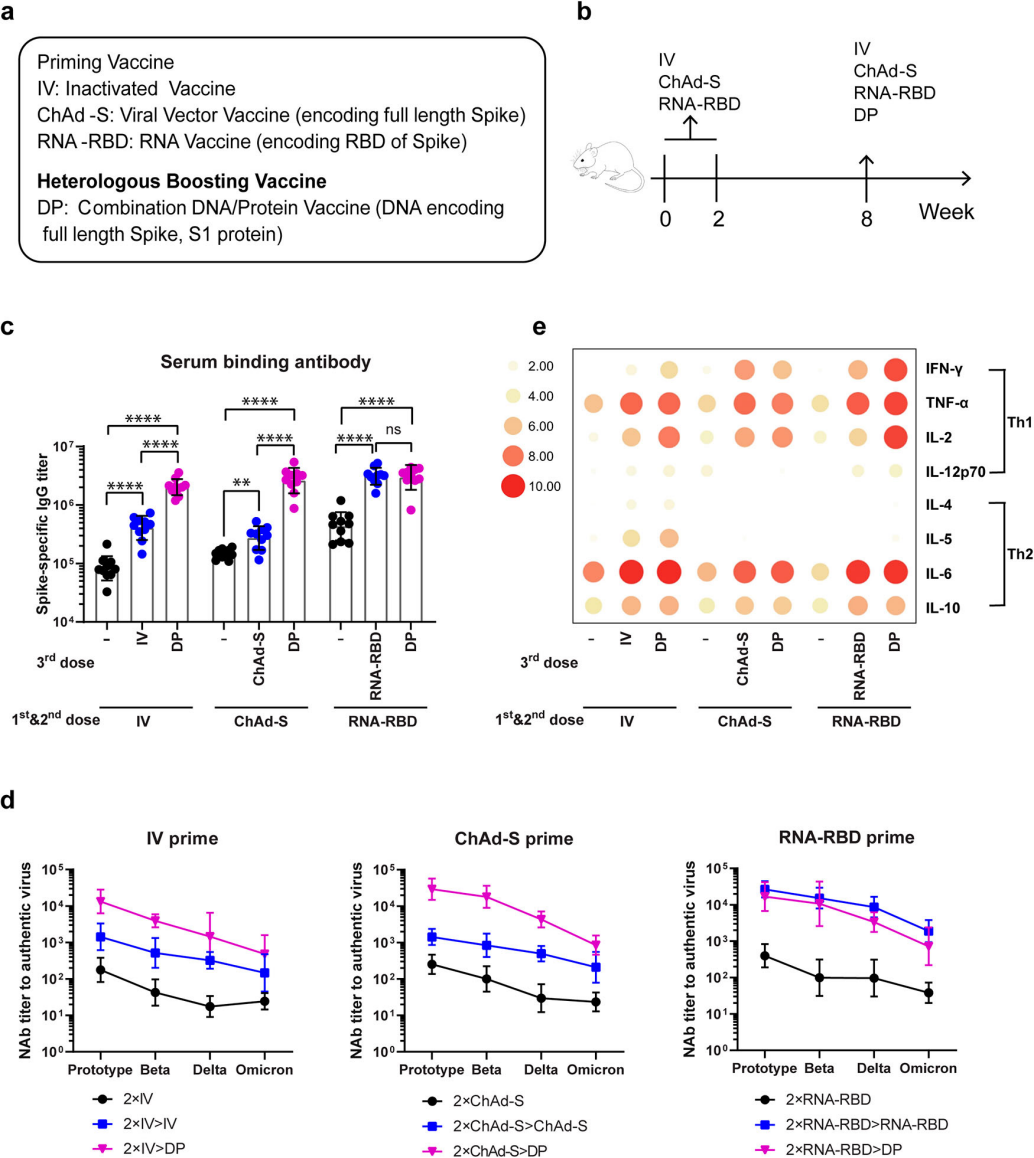
Study design and immune responses. (a). Vaccines used in the study. (b). Mouse immunization schedule *N* = 10 per group. (c). Spike specific IgG titer in mice immune sera as measured by ELISA. (d). Mouse serum Nab titers (50% inhibitory dilution, ID50) against live SARS-CoV-2 prototype virus and variants. (e). T cell immune responses in immunized mice. Mouse splenocytes in different groups were stimulated with full length spike peptide pool and the supernatants were pooled by group for cytokine measurement using MSD. The concentration (pg/mL) of either Th1 (IFN-γ, TNF-α, IL-2, IL-12p70) or Th2 (IL-4, IL-5, IL-6, IL-10) cytokines was transformed by log2 and expressed in a heatmap. The groups marked with symbol “-” in panel c and e indicate that the baseline immune responses for animals who received two doses of priming vaccines without the boost of the third dose of vaccine. Bars represent geomeans ± GSD in panels c and d, **p < 0.01, ****p < 0.0001. Tukey’s multiple comparisons test was used to make comparisons between groups.

Groups of Balb/C mice (6 weeks old) (10 mice per group) first received twice priming vaccines (IV, ChAd-S or RNA-RBD) respectively on Week 0 and Week 2, all by intramuscular needle immunization (IV: 1 μg; ChAd-S: 1 × 10^10^ vp; RNA-RBD: 5 μg) (Figure 1(b)). On Week 8, all animals received one-time boost immunization, either with the same priming vaccines or with heterologous DP vaccine (200 μg DNA/25 μg protein) (Figure 1(b)). Serum samples were collected prior to the first immunization and 2 weeks after each immunization. At the end of study (Week 10), splenocytes were collected for cell immunity analysis.

Serum SARS-CoV-2 spike-specific IgG titers were measured by ELISA. Antibodies after the first two priming immunizations were positive for all immunized mice. The titers in IV and ChAd-S groups were similar, but they are lower than that in RNA-RBD group (Figure 1(c)). After the 3rd dose boost, inactivated vaccine and ChAd-S vaccine were able to significantly boost the antibody titers to animals received the homologous IV or ChAd-S vaccine prime (*p* < 0.0001). But the titers in ChAd-S group were increased modestly after the homologous 3rd dose injection, lower than that in IV group (Figure 1(c)). However, the DP vaccine as the 3rd dose heterologous boost was able to further boost the titers in both IV and ChAd-S primed animal groups to reach a significantly higher level than that achieved by the 3rd dose homologous boost (*p* < 0.0001).

To animals in the RNA-RBD primed group, both homologous RNA-RBD boost and heterologous DP boost achieved the same high-level antibody responses which were significantly higher than the titer before boost (*p* < 0.0001) (Figure 1(c)). Among all groups, heterologous DP boost can raise the antibody levels in both IV and ChAd-S groups to a level similar to that in the RNA-RBD group but the homologous boost with IV or ChAd-S could not do that (Figure 1(c)).

Functional antibody responses were measured by the in vitro neutralizing assay against authentic SARS-CoV-2 viruses (prototype SARS-CoV-2 isolate and its variants Beta, Delta and Omicron (B.1.1.529)) (Figure 1(d)). After twice priming immunizations, all animals developed Nab against both prototype virus and its main variants but the Nab titer was the highest against the prototype virus and decreasing against Beta, Delta and Omicron variants similar as reported in literature. Nab titers are similar in IV and ChAd-S priming groups, and slightly higher in RNA-RBD priming group against either prototype or variants. In IV primed or ChAd-S primed animal groups, homologous boost with either IV or ChAd-S was able to boost the Nab titers against prototype and variants in a pattern very similar to each other, however, heterologous boost with the DP is much more effective to increase Nab titers to all viruses including the variants used in this study.

Boost with either RNA-RBD or DP was able to increase the Nab titers in the RNA-RBD primed animals to the similarly higher levels. It is interesting to see while the Nab titers were low in IV and ChAd-S groups relative to the RNA-RBD group either at the end of priming or after the homologous boost, one-time heterologous boost with DP vaccine was able to increase the Nab titers in both IV and ChAd-S groups to that of the RNA-RBD group. This is true for Nab against either prototype or variants.

T cell immune responses were also measured in the current study. Animal splenocyte were collected and the expression of Th1 (IFN-γ, TNF-α, IL-2 and IL-12p70) and Th2 (IL-4, IL-5, IL-6 and IL-10) cytokines were measured after stimulation with peptide pools spanning the SARS-CoV-2 spike protein. After the twice priming immunizations, overall the T cell immune responses were low with the exception of TNF-α and IL-6 and IL-10 (Figure 1(e)). Homologous boost with vaccines matching with the priming ones or heterologous boost with DP vaccine had similar effects with increased levels of TNF-α and IL-6 and IL-10. But boost with DP vaccine was more effective with increased levels of IL-2 and IFN-γ. IL-5 was only detectable in IV group, after either IV or DNA/protein boost. It is interesting to observe heterologous DP boost was more effective than homologous RNA-RBD in boosting multiple cytokines.

In summary, the current study confirms that the combination DNA/ Protein COVID-19 formulation as previously reported is highly immunogenic and can be used as a booster vaccine to hosts who received a wide range of priming vaccines including mRNA vaccines. Such boosting is effective in overall antibody titers, Nab titers and T cell immune responses. While combination DNA/Protein vaccine boost may not be more effective than mRNA vaccine boost, it provides an alternative option in addition to the mRNA vaccine. We did not dissect the components of the combination DNA/protein vaccine boost to understand to what degree that each component was contributing to the boosting effects observed in this mouse pilot study. More advanced studies are warranted especially in non-human primate models and human beings to include such analysis to further optimize the heterologous prime-boost formulation, as part of the strategy in developing the next generation COVID-19 vaccines with enhanced and long-lasting immunity.

## Supplementary Material

Supplemental Material
