# Supplementary material for: Mouse study of combined DNA/protein COVID-19 vaccine to boost high levels of antibody and cell mediated immune responses
Source: Emerg Microbes Infect. 2022 Dec 12;12(1):2152388. doi: 10.1080/22221751.2022.2152388 (PMC9754044; doi:10.1080/22221751.2022.2152388)
Supplement: Supplemental Material [file TEMI_A_2152388_SM9108.docx]

**Supplementary materials**

**Table S1. The spike specific IgG titers induced by different vaccination regimen and the data were used to plot Figure 1c (n=10/group).**

| Mouse no. | Spike specific IgG titers | | | | | | | | | | | |
| --- | --- | --- | --- | --- | --- | --- | --- | --- | --- | --- | --- | --- |
|  | Prime with 2xIV | | |  | Prime with 2xChAd-S | | |  | Prime with 2xRNA | | | |
|  | 2xIV | 2xIV  >IV | 2xIV  >PD |  | 2xChAd-S | 2xChAd-S  >ChAd-S | 2xChAd-S  >PD |  | 2xRNA-RBD | 2xRNA-RBD  >RNA-RBD | 2xRNA-RBD  >PD |  |
| 1 | 106000 | 730476 | 3580952 |  | 191429 | 521905 | 3438095 |  | 1190476 | 4695238 | 3228571 |  |
| 2 | 64571 | 242857 | 2885714 |  | 168571 | 168571 | 874286 |  | 359048 | 3180952 | 4247619 |  |
| 3 | 89810 | 517143 | 2047619 |  | 178095 | 301905 | 1609524 |  | 647619 | 3028571 | 4409524 |  |
| 4 | 32762 | 613333 | 2019048 |  | 142857 | 115238 | 3685714 |  | 220952 | 2647619 | 2628571 |  |
| 5 | 214286 | 144762 | 1390476 |  | 112381 | 172381 | 2685714 |  | 525714 | 2323810 | 3542857 |  |
| 6 | 111429 | 348571 | 2142857 |  | 167619 | 376191 | 2495238 |  | 477143 | 3314286 | 2676190 |  |
| 7 | 78571 | 379048 | 1761905 |  | 148571 | 329524 | 2257143 |  | 637143 | 3400000 | 3628571 |  |
| 8 | 63333 | 540000 | 2180952 |  | 140952 | 319048 | 5438095 |  | 234286 | 5180952 | 4047619 |  |
| 9 | 75429 | 446667 | 1190476 |  | 128571 | 256191 | 3200000 |  | 462857 | 1590476 | 820000 |  |
| 10 | 80000 | 466667 | 1952381 |  | 108571 | 410476 | 3238095 |  | 211429 | 3219048 | 2800000 |  |

**Table S2. The geometric mean of neutralizing antibody titers against different viruses (n=10/group) and the data were used to plot Figure 1d including the statistical significance for the difference between the PD boost and the homologous boost groups.**

| SARS-Cov-2 variants | Nab titers | | | | P value* |
| --- | --- | --- | --- | --- | --- |
|  | 2xIV | Homologous boost |  | PD boost |  |
|  |  | 2xIV>IV |  | 2xIV>PD |  |
| Prototype | 177 | 1424 |  | 13239 | <0.0001 |
| Beta | 43 | 515 |  | 3913 | 0.0001 |
| Delta | 17 | 323 |  | 1441 | 0.0155 |
| Omicron | 24 | 146 |  | 481 | 0.1337 |
|  |  |  |  |  |  |
| SARS-Cov-2 variants | Nab titers | | | | P value* |
|  | 2xChAd-S | Homologous boost |  | PD boost |  |
|  |  | 2xChAd-S > ChAd-S |  | 2xChAd-S>PD |  |
| Prototype | 252 | 1424 |  | 29206 | <0.0001 |
| Beta | 100 | 837 |  | 17978 | <0.0001 |
| Delta | 30 | 495 |  | 4316 | <0.0001 |
| Omicron | 23 | 209 |  | 847 | 0.0009 |
|  |  |  |  |  |  |
| SARS-Cov-2 variants | Nab titers | | | | P value* |
|  | 2xRNA-RBD | Homologous boost |  | PD boost |  |
|  |  | 2xRNA-RBD > RNA-RBD |  | 2xRNA-RBD > PD |  |
| Prototype | 397 | 26478 |  | 16775 | 0.9993 |
| Beta | 99 | 15207 |  | 10627 | 0.9992 |
| Delta | 97 | 8587 |  | 3337 | 0.5089 |
| Omicron | 38 | 1879 |  | 720 | 0.4443 |

*****Statistical significance between PD boost and homologous boost groups

**Table S3. The average concentration of cytokines data used to plot Figure 1e (pg/ml) (n=10/group).**

| Cytokines | Concentration of cytokines (pg/ml) | | | | | | | | | | |
| --- | --- | --- | --- | --- | --- | --- | --- | --- | --- | --- | --- |
|  | Prime with 2xIV | | |  | Prime with 2xChAd-S | | |  | Prime with 2xRNA-RBD | | |
|  | 2xIV | 2xIV  >IV | 2xIV  >PD |  | 2xChAd-S | 2xChAd-S  >ChAd-S | 2xChAd-S  >PD |  | 2xRNA-RBD | 2xRNA-RBD  > RNA-RBD | 2xRNA-RBD  > PD |
| IFN-γ | 0.03 | 3.77 | 40.6 |  | 1.7 | 127.54 | 65.39 |  | 0.42 | 82.64 | 534.44 |
| IL-10 | 31.52 | 80.85 | 86.58 |  | 20.21 | 57.25 | 53.2 |  | 18.81 | 93.36 | 80.58 |
| IL-12p70 | 0.65 | 2.99 | 5.11 |  | 4.94 | 0 | 0.58 |  | 0 | 5.87 | 8.52 |
| IL-2 | 2.4 | 59.76 | 235.12 |  | 8.72 | 107.68 | 149.41 |  | 7.36 | 47.17 | 629.15 |
| IL-4 | 0.24 | 2.92 | 3.46 |  | 0.01 | 0.12 | 0.16 |  | 0 | 0.04 | 0.53 |
| IL-5 | 1.16 | 36.51 | 70.23 |  | 0.03 | 0.32 | 0.09 |  | 0 | 0.12 | 1.15 |
| IL-6 | 192.39 | 902.63 | 964.7 |  | 79.06 | 391.04 | 415.07 |  | 40.98 | 754.88 | 794.09 |
| TNF-α | 64.37 | 340.09 | 333.11 |  | 44.43 | 306.37 | 226.28 |  | 35.82 | 408.61 | 602.93 |
